# Identification of Candidate Genes and MicroRNAs for Acute Myocardial Infarction by Weighted Gene Coexpression Network Analysis

**DOI:** 10.1155/2019/5742608

**Published:** 2019-02-11

**Authors:** Yan Li, Xiao_nan He, Chao Li, Ling Gong, Min Liu

**Affiliations:** ^1^Department of Epidemiology and Biostatistics, School of Public Health, Peking University, Beijing, China; ^2^Department of Epidemiology, Beijing An Zhen Hospital, Capital Medical University, Beijing Institute of Heart, Lung and Blood Vessel Diseases, Beijing, China; ^3^Emergency and Critical Care Center, Beijing An Zhen Hospital, Capital Medical University, Beijing, China; ^4^Cardiovascular Center, Beijing Tongren Hospital, Capital Medical University, Beijing, China

## Abstract

**Background:**

Identification of potential molecular targets of acute myocardial infarction is crucial to our comprehensive understanding of the disease mechanism. However, studies of gene coexpression analysis via jointing multiple microarray data of acute myocardial infarction still remain restricted.

**Methods:**

Microarray data of acute myocardial infarction (GSE48060, GSE66360, GSE97320, and GSE19339) were downloaded from Gene Expression Omnibus database. Three data sets without heterogeneity (GSE48060, GSE66360, and GSE97320) were subjected to differential expression analysis using MetaDE package. Differentially expressed genes having upper 25% variation across samples were imported in weighted gene coexpression network analysis. Functional and pathway enrichment analyses were conducted for genes in the most significant module using DAVID. The predicted microRNAs to regulate target genes in the most significant module were identified using TargetScan. Moreover, subpathway analyses using iSubpathwayMiner package and GenCLiP 2.0 were performed on hub genes with high connective weight in the most significant module.

**Results:**

A total of 1027 differentially expressed genes and 33 specific modules were screened out between acute myocardial infarction patients and control samples. Ficolin (collagen/fibrinogen domain containing) 1 (*FCN1*), CD14 molecule (*CD14*), S100 calcium binding protein A9 (*S100A9*), and mitochondrial aldehyde dehydrogenase 2 (*ALDH2*) were identified as critical target molecules;* hsa-let-7d*,* hsa-let-7b*,* hsa-miR-124-3*, and* hsa-miR-9-1* were identified as potential regulators of the expression of the key genes in the two biggest modules.

**Conclusions:**

* FCN1*,* CD14*,* S100A9*,* ALDH2*,* hsa-let-7d*,* hsa-let-7b*,* hsa-miR-124-3,* and* hsa-miR-9-1* were identified as potential candidate regulators in acute myocardial infarction. These findings might provide new comprehension into the underlying molecular mechanism of disease.

## 1. Introduction

Acute myocardial infarction (AMI) is characterized by definite evidence of myocardial necrosis in a clinical background of acute myocardial ischemia [1]. According to the report of global burden of cardiovascular disease in 2015, there were an estimated 7.29 million AMI in the world [2], which contributed to a high morbidity and mortality of global health. AMI has a high risk of death due to congestive heart failure and malignant arrhythmia, although many endeavors and monies have been spent on developing new therapies, it remains a major challenge for clinicians to prevent the adverse cardiac events and cure this disease. Established risk factors, such as hypertension, diabetes, hypercholesterolemia, smoking, and obesity, cannot explain all the risk on morbidity and mortality of AMI, and, moreover, substantial numbers of patients have an inexplicit etiology in myocardial damage [3]. Therefore, urgent research to discover potential pathogenesis of AMI and exploit novel medication targets and therapeutic strategies is needed, thereby reducing the threat of this disease to human life.

The clinical manifestations and characteristics of AMI are heritable features, and transcriptomics-based screening of genetic biomarker is helpful in early recognition of risk carriers and improving diagnosis and treatment of AMI. It is a general recognition that expression microarrays have advantages of rapid unbiased screening and extensive coverage of nearly all transcriptomes to reveal the most promising targets. IL-1RL-1, Interleukin 1 receptor-like 1 (ST2) should be a representation of cardiac biomarker which was identified as a target at initial microarray analyses and then resulted in the development of suitable assay [4]. Recently, microRNAs (miRNAs) as noncoding small RNAs are involved in a broad range of regulation for biological processes and disease development, and also several evidences have shown that circulating miRNAs are stable and can be used as novel diagnostic markers for AMI [5–10].

Coexpression networks as transcriptomic technologies have grown in popularity since they allow for the integration of large transcriptional data sets, as well as coinstantaneous identification, clustering and analysis of thousands of genes with similar expression patterns across a wide range of conditions [11]. Weighted Gene Coexpression Network Analysis (WGCNA) has been established by means of introducing several adjacency functions that convert the coexpression measure to a connection weight, and furthermore the parameters of the adjacency function have been determined by the scale-free topology criterion [12]. WGCNA provides a powerful network-based strategy to expedite the clarification of molecular mechanisms underlying important biological processes and applies to a diverse range of human disease researches. Accordingly, WGCNA could be used to analyze AMI microarray data sets in this study.

So far gene expression studies of AMI have been limited in sample size and lack of myocardial tissue samples from patients. On the other hand, development and utility of circulating biomarkers would be optimum to noninvasive diagnosis and early identification of AMI. We combined several blood-based microarray expression data to screen out mutual differentially expressed genes (DEGs) among data sets. WGCNA was used to construct gene coexpression network based on DEGs profiling, and significant modules and hub genes were detected by the WGCNA as well. Furthermore, miRNAs that could be predicted to regulate DEGs in the most significant module were identified and miRNAs-DEGs regulatory relationships were analyzed. This study aimed to detect out more candidate genes and miRNAs that were involved in the pathogenesis and progression of AMI, among which some genetic biomarkers might be converted into the promising targets for the diagnosis and treatment of AMI.

## 2. Materials and Methods

### 2.1. Source of Microarray Data

Four microarray data, including GSE48060 (USA; 31 patients with AMI and 21 controls), GSE66360 (USA; 49 patients with AMI and 50 controls), GSE97320 (China; 3 patients with AMI and 3 controls), and GSE19339 (Switzerland; 4 patients with AMI and 4 controls), were downloaded from Gene Expression Omnibus (GEO http://www.ncbi.nlm.nih.gov/geo/) database, which were based on the platform of GPL570 Affymetrix Human Genome U133 Plus 2.0 Array (Affymetrix Inc., Santa Clara, California, USA). This microarray meta-analysis made use of four data sets based on 156 blood samples derived from 87 AMI patients and 78 control subjects.

### 2.2. Data Preprocessing

The raw array data (CEL files) were imported into Expression Console software and subjected to background adjustment, quantile normalization, and log_2_ transformation by Robust Multiarray Average (RMA) [13]. After that, probe identifiers (IDs) were transformed into gene symbols according to the annotation files, and the average expression value of multiple probes corresponding to one same gene was calculated as the single expression value of this gene.

### 2.3. Quality Control of Microarray Data and Differential Expression (DE) Analysis

Six quality control (QC) indices provided in MetaQC package of R software were used to assess quality of selected studies, which included internal homogeneity of coexpression structure among studies (IQC), external consistency of coexpression pattern with pathway database (EQC), accuracy and consistency of differentially expressed gene detection (AQCg and CQCg), and enriched pathway identification (AQCp and CQCp) [14]. By applying this procedure, we could filter out the low-quality studies and eliminate the biases among datasets. Moreover, principal component analysis (PCA) biplots and standardized mean ranks were provided assisting in the detection of deflected studies. MetaDE package in R software provided 12 major statistical methods for differential expression (DE) analysis [14]. We chose DEGs between disease group and control group using the MetaDE.pvalue algorithm of MetaDE package. A false discovery rate (FDR) of < 0.05 was considered as the cut-off for the detection of DEGs.

### 2.4. Gene Ontology (GO) and Pathway Enrichment Analysis

Gene ontology (GO) is a tool for gene annotation using a dynamic, controlled vocabulary that classifies genes into three categories, including biological process, molecular function, and cellular component [15]. Kyoto Encyclopedia of Genes and Genomes (KEGG) database is used to assign gene sets to specific pathway maps of molecular interactions, reactions, and relation networks [16]. We performed GO functional annotation and KEGG pathway enrichment analyses of DEGs by the Database for Annotation, Visualization and Integrated Discovery (DAVID) [17].

### 2.5. Weighted Gene Coexpression Network Construction

We conducted gene coexpression analysis of DEGs in order to check out more potential genes associated with AMI. For reducing the deviation, we chose DEGs having upper 25% variation across samples to implement subsequent weighted gene coexpression network analysis (WGCNA).

WGCNA was used to construct weighted adjacency matrix that reported the connection strength between gene pairs. For weighted networks, the concordance of gene expression was measured with the Pearson correlation matrix, and the absolute value of the correlation coefficient *s*_*ij*_ was calculated as *s*_*ij*_ = |*cor*(*x*_*i*_, *x*_*j*_)|, where *x*_*i*_ and *x*_*j*_ were the vectors of the expression value of gene* i* and gene* j*. Then, the Pearson correlation matrix was transformed continuously with the power adjacency function into weighted gene network, and the adjacency matrix *a*_*ij*_ was calculated as  *a*_*ij*_ = *s*_*ij*_^*β*^. Here, the exponential *β* = 20 were chosen by scale-free topology criterion (Supplementary Figure 1). For detecting gene coexpression modules, the adjacency matrix was converted into the topological overlap matrix (TOM). The following formula was used to compute the weighting coefficient *W*_*ij*_ (referred to TOM):

(1) Wij=lij+aijmin⁡ki,kj+1−aijlij=∑uaiuaujki=∑uaiu

where *k*_*i*_ indicated the total connectivity of gene* i* with all other genes in the weighted network, *W*_*ij*_ was considered the topology overlap between neighbor genes of* i* and* j*. The topological overlap dissimilarity (1 – TOM) was used as input of hierarchical clustering. Modules were defined as branches of a hierarchical clustering dendrogram using the average linkage hierarchical clustering coupled with the topological overlap dissimilarity measure. For each module, the module eigengene (ME) was summarized by the first principal component of the module expression levels, and the correlation between each gene expression values and module eigengene was defined as the module membership (MM). A gene significance (GS) was measured as minus log of a* p*-value with the* T*-test. The module significance (MS) was determined as the mean absolute GS for all genes in a given module.

### 2.6. Module Preservation Statistics

Module preservation statistics are used to verify whether an identified module in the reference network can be found in the test network. We investigated the preservation of coexpression network between the reference data set (meta-analysis) and the test data set (GSE123487) using the network-based Zsummary calculated by the module preservation function from WGCNA [18]. The statistics are calculated twice: once to evaluate whether modules are reproducible in the reference data set consisting only of genes in common with the test data set (called* Zsummary.qual* as “quality” statistics) and the second time to assess the conservation of the modules in the test data set (called* Zsummary.pres* as “preservation” statistics).* Zsummary.pres* < 2 implies no evidence for module preservation, 2 <* Zsummary.pres* < 10 implies weak to moderate evidence of preservation, and* Zsummary.pres* > 10 implies strong evidence that the module is preserved.* Zsummary.qual* was a complementary statistic to evaluate the robustness of the identified modules.

### 2.7. Screening of Hub Genes and Subpathway Analysis

In general, the genes with the largest number of connections are the most important genes in a module. The hub gene is termed as an abbreviation of “highly connected gene” that tends to have high connectivity in a coexpression module. Additionally, the hub genes were described as the genes most closely associated with disease. Intramodular connectivity (IC) corresponds to the connection degree of a gene with other genes in a given module. Here, the hub genes were considered as the highly connected genes according to their characterization with high IC, high MM, and high GS. After screening of the hub genes, a web-based text-mining server of GenCLiP 2.0 (http://ci.smu.edu.cn/GenCLiP2/analysis.php) was used to identify biological functions and molecular interactions in the hub genes list. The iSubpathwayMiner provides the k-clique method for identification of metabolic subpathways associated with studying disease based on the interested gene sets [19]. We used iSubpathwayMiner in mining the most relevant metabolic subpathways of the hub genes by merging information from genes and metabolites. The significantly enriched subpathways were detected by hypergeometric test (*p*-value < 0.05).

### 2.8. MicroRNAs (miRNAs) Target Prediction

To identify putative miRNA binding sites at the 3'-untranslated regions (3'-UTR) of mRNAs, we conducted miRNAs target prediction using the TargetScanHuman 7.2 algorithm. The outputs of prediction are ranked based on either the predicted efficacy of targeting (context++ scores) [20] or the probability of conserved targeting (P_CT_) [21]. Here, the combination of two measures on context score percentile ≥ 90 and aggregate P_CT_ score ≥ 0.8 was used to determine effective miRNAs. The predicted results of miRNAs were validated with data set of GSE123487.

## 3. Results

### 3.1. Screening of DEGs

**Figure 1:**
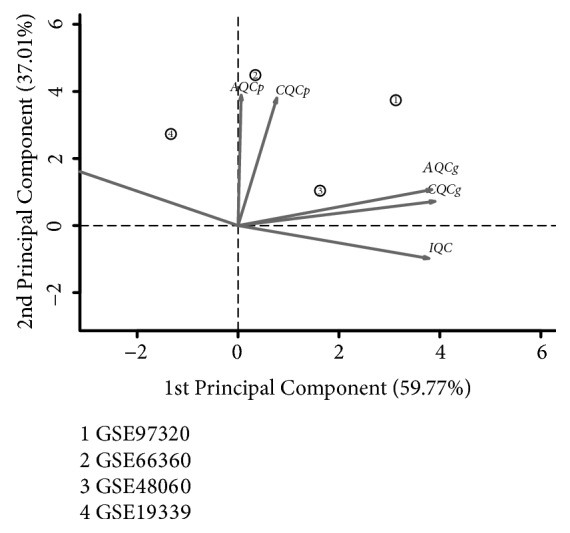
Principal component analysis biplots of quality control measures in four microarray data sets.

In order to eliminate problematic study and obtain reliable data, we performed MetaQC analysis of four data sets (GSE48060, GSE66360, GSE97320, and GSE19339) downloaded from GEO database. Six QC indexes and the standardized mean rank summary (SMR) scores were created by R software. The QC measures indicated that a study (GSE19339) had relatively low correlation with other three studies (Table 1). Coupled with the visualization of PCA biplots describing that a study on the opposite side of arrows had large SMR scores (Figure 1), the data set of GSE19339 was finally excluded from meta-analysis. DEGs were selected by MetaDE analysis. A total of 1027 DEGs were identified between AMI group and control group under the threshold of FDR < 0.05 (Supplementary Figure 2).

### 3.2. WGCNA Network Construction and Key Modules Detection

**Figure 2:**
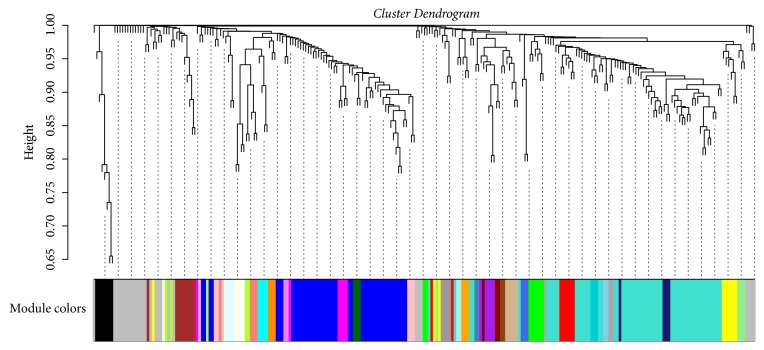
The clustering dendrogram for identification of gene coexpression modules in AMI by using the average linkage hierarchical clustering.

One hundred and fifty-seven samples with disease phenotypes were involved in gene coexpression analysis. For reducing the deviation, we selected DEGs having upper 25% variation across samples to implement WGCNA. To construct a weighted network, the lowest power of *β* = 20 (R^2^ ≥ 0.8) was chosen for a criterion of scale-free topology. By the means of the average linkage hierarchical clustering, two hundred and fifty-seven DEGs were grouped into a total of 33 modules (Figure 2). We selected the first largest module (56 DEGs clustered in turquoise module) and the second largest module (45 DEGs clustered in blue module) as the key (interesting) modules for further analysis. For each key module, the MS value was used to test their association with the disease. That the turquoise module had a higher MS value (MS_turquoise_ = 5.71) than the blue module (MS_blue_ = 2.64) suggested that the turquoise module had stronger correlation to AMI. In addition, the relevance between ME per key module and the disease status was also measured via calculation of Pearson's correlation coefficient and the significant* p*-value. The turquoise module (r = 0.40,* p*-value = 1.73E-07) was still considered to be highly relevant to AMI than the blue module (r = -0.27,* p*-value = 7.35E-04) (Supplementary Figure 3). We conducted statistical comparisons of correlations between the turquoise module and the blue module with AMI. The calculations relied on the tests implemented in the package* cocor* for the R programming language. The results of a comparison between two correlations showed that there was statistically significant difference (z=4.71, p-value<0.001). Subsequently, we conducted functional and pathway enrichment analysis for all DEGs clustered in the turquoise module. The mainly enriched results of significant functions and pathways were described in Table 2.

### 3.3. Identification of Hub Genes Associated with AMI

**Figure 3:**
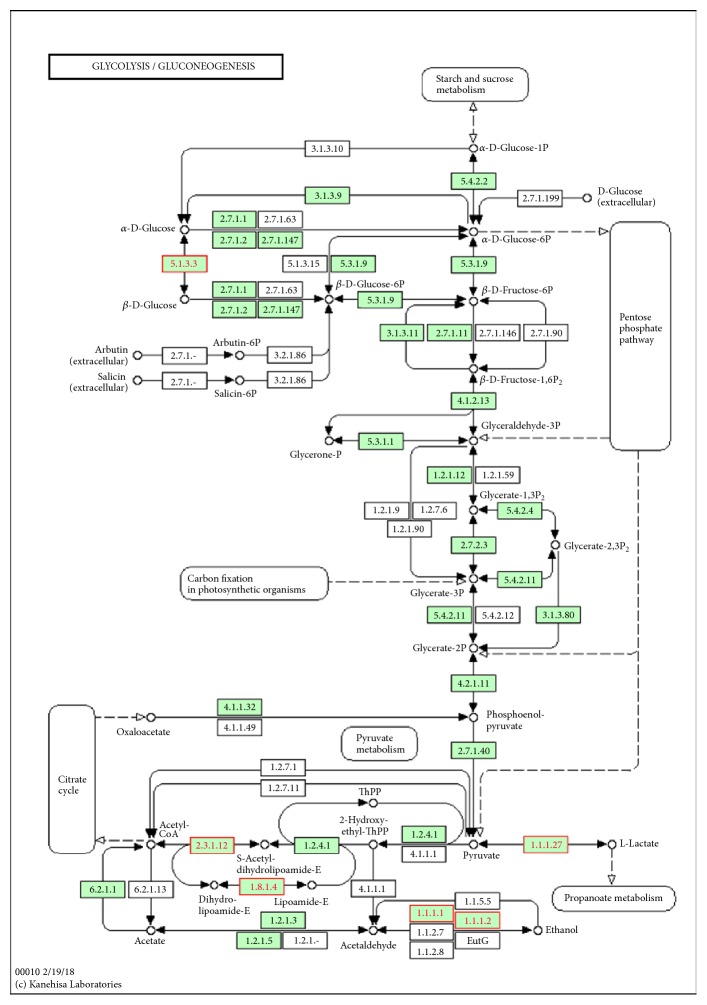
*ALDH2* enriched in subpathway from regulation of glycolysis/gluconeogenesis.

To identify intramodular hub genes, we computed the IC, MM, and GS for each gene in the interesting module. Through comparing with these measures, we found a positive correlation between MM and IC, but not GS. So, we adopted the IC and MM values as selective measures of the hub genes for each of the key modules (Table 3). The top hub genes in the turquoise module included* FCN1*,* S100A9*,* IGSF6*,* HCK*,* CD14*,* TLR2*,* VCAN*,* PTAFR*,* GLT1D1,* and* MS4A6A*, and the top hub genes in the blue module included* ABCA5*,* LUC7L*,* RBM6*,* FAM134B*,* CLUAP1,* and* GABPB2*. The iSubpathwayMiner can detect the local structure of an entire pathway (subpathway) that will help us to understand the pathogenic mechanism caused by dysfunction of the subpathways. For the turquoise module, the subpathway analysis displayed that* ALDH2* gene was enriched in the subpathway of glycolysis/gluconeogenesis (Figure 3). Furthermore, all highly connective genes (adjacency weight greater than 0.1) of the turquoise module were submitted to GenCLiP 2.0 for data mining. The results showed that the hub genes in the network enriched in several functional terms of biological processes, including regulation of response to wounding and regulation of phosphate metabolic process.

### 3.4. Replication of Module Structure and Hub Genes

We tested the module preservation of the reference data set (meta-analysis) in the validation data set (GSE123487). Among 33 identified modules, we found there was low-to-moderate evidence of preservation (*Zsummary.pres* = 3.5) for the turquoise module; however, other modules were not preserved (*Zsummary.pres* < 2). To further confirm the findings of intramodular hub genes, we performed differentially expressed mRNAs analysis in the test data set. The hub genes* FCN1* (*p*-value = 0.002),* HCK* (*p*-value = 0.012),* CD14* (*p*-value = 0.024),* TLR2* (*p*-value = 0.009),* VCAN* (*p*-value = 0.009), and* GLT1D1* (*p*-value = 0.022) in the turquoise module identified by the reference data set, as well as the hub genes* ABCA5* (*p*-value = 0.0004),* LUC7L* (*p*-value = 0.013), and* GABPB2* (*p*-value = 0.039) in the blue module, were also found to be differentially expressed in the test data set.

### 3.5. MiRNAs Target Prediction and Validation

**Figure 4:**
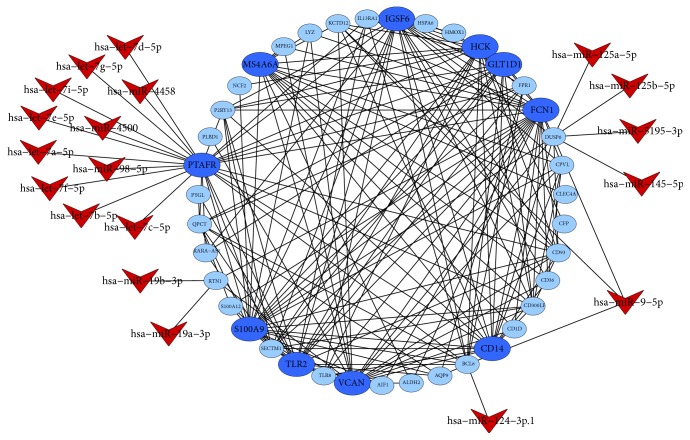
The putative miRNAs regulatory network for DEGs of turquoise module.

**Figure 5:**
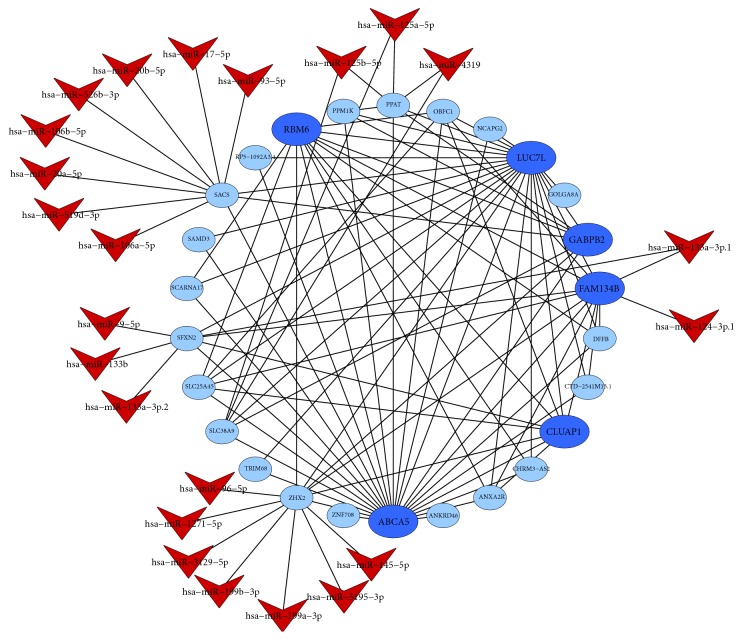
The putative miRNAs regulatory network for DEGs of blue module.

TargetScan was applied to predicate the miRNAs regulated to the target DEGs in the network of key module. Only a context score percentile ≥ 90 and an aggregate P_CT_ score ≥ 0.8 were identified as the putative miRNA of the target gene (Figures 4 and 5). The main results represented that the target genes* PTAFR*,* BCL6*,* DUSP6,* and* KCTD12* in the turquoise module were regulated by* hsa-let-7-5p*,* hsa-miR-124-3p.1*,* hsa-miR-145-5p,* and* hsa-miR-9-5p*, respectively. Moreover,* hsa-miR-124-3p.1* and* hsa-miR-9-5p* also regulated the target genes* FAM134B* and* SFXN2* in the blue module. The predicted results of miRNAs were tested with data set of GSE123487 using microarray assays. There were* hsa-miR-9-1* (*p*-value = 0.026),* hsa-miR-124-3* (*p*-value = 0.046),* hsa-miR-5195* (*p*-value = 0.014),* hsa-let-7d* (*p*-value = 0.028),* hsa-let-7b* (*p*-value = 0.026),* hsa-miR-4500* (*p*-value = 0.024),* hsa-miR-4319* (*p*-value = 0.037),* hsa-miR-133b* (*p*-value = 0.040),* hsa-miR-526b* (*p*-value = 0.036), and* hsa-miR-5195* (*p*-value = 0.014) that were confirmed to be differentially expressed between AMI patients and normal controls.

## 4. Discussion

This study aimed to identify candidate genes and miRNAs involved in the occurrence and development of AMI. In this study, we screened out total of 1027 DEGs in blood samples from AMI patients compared with normal controls. The average linkage hierarchical clustering analysis was carried out to group coexpressed DEGs into modules, and 33 modules were identified. Among them, the turquoise and blue modules were detected as the two biggest modules to be associated with AMI. From the functional enrichment and GO analysis, we found that DEGs were mainly correlated to functional annotations of inflammatory and immune responses. The subpathway enrichment analysis revealed that* ALDH2* gene in the turquoise module was enriched in glycolysis/gluconeogenesis subpathway. Furthermore,* hsa-let-7d*,* hsa-let-7b*,* hsa-miR-124-3*, and* hsa-miR-9-1* were identified to regulate the key genes in the turquoise and blue modules.

Myocardial ischemia causes a reduction of oxygen supply to the heart, leading to cardiac myocytes in the hypoxia status dependent of the glycolysis metabolism, the major source of energy supply in the hypoxic circumferences. In spite of the fact that cardiovascular drugs and surgical interventions can improve the oxygen supply in myocardium for increased cardiac work, these interventions were shown to be unsatisfactory to decrease cardiac events or increase patient survival [22]. Aldehydes have been reported to be highly associated with myocardial ischemia and cardiac reperfusion damage [23, 24]. Here, subpathway analysis found that* ALDH2* might exert its role by influencing glycolysis/gluconeogenesis metabolic pathway. Mitochondrial aldehyde dehydrogenase 2 (*ALDH2*) is an important enzyme that catalyses the removal of reactive aldehydes, whose activation was indicated to be correlated with reduced ischemic myocardium damage in rodent models [25]. Acetaldehyde metabolites, such as 4-hydroxynonenal (*4-HNE*), can affect glycolysis by modifying key glycolytic enzymes, including glyceraldehyde-3-phosphate dehydrogenase and glucose-6-phosphate dehydrogenase [26], and also they can inhibit mitochondrial respiratory chain function, promote mitochondrial membrane permeability transformation channel open, and directly lead to mitochondrial dysfunction. Therefore,* ALDH2* confers profound treatment-aided value in individualized cardioprotective strategies.

The genes in turquoise module, including* HMOX1*,* TLR2*,* VCAN*,* S100A9*,* CD14,* and* CD36*, were significantly correlated to wound healing function; in addition,* HCK*,* HMOX1*,* BCL6*,* S100A9*,* S100A12*,* TLR2*, and* CD36* were significantly enriched in response to wounding associated biological process. It is well established that myocardial infarction is accompanied by local and systemic inflammation and leads to rapid necrosis of myocardia in the ischemic heart [27]. For postmyocardial infarction, healing the myocardium wound is essential for tissue integrity and function of the heart. There is widespread concern and increased interest in the damage associated molecular pattern molecules* S100A8* and* S100A9* in human cardiovascular disease.* S100A8* and its binding partner* S100A9* are members of the S100 calcium-binding family of proteins, the circulating levels of which are elevated by activated inflammatory cytokines and autoimmune state [28]. The growing evidence indicated that* S100A9* played an important role in leukocyte trafficking and arachidonic acid metabolism [29, 30]. In human, several* S100* proteins, including* S100A7*,* S100A8*,* S100A9*, and* S100A12,* are linked with the severity of coronary and carotid atherosclerosis [31–34]. Thus,* S100A8* and* S100A9* might serve as a useful biomarker and therapeutic target in human cardiovascular disease; besides,* S100A8* and* S100A9* blockers have been developed and are approved for clinical testing.

Some DEGs clustered in the turquoise module have been reported to be associated with AMI, including* S100A9* [35],* CD14* [36],* TLR2* [37], and* HMOX1* [38]. However, human studies on the association between* FCN1* and AMI have not been reported, although there was a study in mice that MBL/ficolin-associated protein-1 (*MAP-1*) can attenuate myocardial injury and arterial thrombogenesis [39]. We analyzed the interactional associations of proteins encoded by DEGs in the turquoise module using the Search Tool for the Retrieval of Interacting Genes/Proteins (STRING; https://string-db.org/). The results showed that there were complicated interactions among* TLR2*,* HMOX1*,* MMP9*,* S100A9*,* CD14*, and* FCN1*. Therefore, the modules identified in the present study associated with AMI could not be considered to be independent of already established genes.

In this study, several miRNAs were predicted to regulate the key genes that contributed to the pathophysiological consequences of AMI. The* let-7* is the second miRNA found in* C.elegans*, and it is highly expressed in the cardiovascular system [40], exerting important regulatory roles after myocardial infarction [41]. There were* hsa-let-7d* and* hsa-let-7b* confirmed to be differentially expressed between AMI patients and normal controls in the validation data set, suggesting that they might play a similar role in regulated process of AMI. Clinical data revealed that circulating* miR-124* and* miR-145* was significantly associated with AMI [42, 43], and also* miR-145* was correlated with the severity of coronary artery [44] and played roles in regulating the evolution of atherosclerotic plaque toward instability and rupture [45]. Besides that,* miR-9* was also found to be probably involved in myocardial regeneration [46].

However, some limitations should be reinforced in this study. Firstly, majority of data derives from two large studies in the USA. Therefore the results are not surely applicable to other ethnicities. Secondly, some findings need to be further studied. Despite these limitations, this study still provides some novel viewpoints in current understanding of AMI mechanism.

## 5. Conclusions

In summary, we screen out candidate genes for AMI, such as* FCN1*,* CD14*,* S100A9*, and* ALDH2*; in addition, we identified* hsa-let-7d*,* hsa-let-7b*,* hsa-miR-124-3,* and* hsa-miR-9-1*, as potential regulators on pathogenesis of AMI. Thus this study may offer potential therapeutic targets and new therapeutic strategies for AMI.

## Figures and Tables

**Table 1:** Microarray data information and quality control measure summary.

GEO accession	Platform	Probe number	Country	AMI sample	Control sample	IQC	EQC	CQCg	CQCp	AQCg	AQCp	SMR
GSE97320	GPL570 / Affymetrix HU133 Plus 2.0	54,676	China	3	3	5.62	2.92	41.88	65.55	27.32	22.53	1.67
GSE66360	GPL570 / Affymetrix HU133 Plus 2.0	54,676	USA	49	50	3.30	4.00	17.76	59.30	16.73	34.49	2.08
GSE48060	GPL570 / Affymetrix HU133 Plus 2.0	54,676	USA	31	21	5.62	2.92	22.33	12.49	16.70	3.55	3.00
GSE19339	GPL570 / Affymetrix HU133 Plus 2.0	54,676	Switzerland	4	4	1.30*∗*	4.00	4.92	39.73	4.49	13.15	3.25

GEO: Gene Expression Omnibus; *∗* represents nonstatistical significance of quality control measures; AMI: acute myocardial infarction; IQC: internal quality index; EQC: external quality index; CQCg and CQCp: consistency quality control indexes; AQCg and AQCp: accuracy quality control indexes; SMR: standardized mean rank.

**Table 2:** Top significantly GO function and KEGG pathway enrichment analysis in the turquoise module.

Category	ID	Term	Count	*p*-value
GO_BP	GO:0006954	inflammatory response	11	7.50E-08
GO_BP	GO:0038124	toll-like receptor TLR6:TLR2 signaling pathway	3	4.58E-05
GO_BP	GO:0071726	cellular response to diacyl bacterial lipopeptide	3	4.58E-05
GO_BP	GO:0031663	lipopolysaccharide-mediated signaling pathway	4	9.63E-05
GO_BP	GO:0002755	MyD88-dependent toll-like receptor signaling pathway	4	1.06E-04
GO_BP	GO:0006955	immune response	8	1.57E-04
GO_BP	GO:0045087	innate immune response	8	1.78E-04
GO_BP	GO:0071223	cellular response to lipoteichoic acid	3	2.73E-04
GO_BP	GO:0032760	positive regulation of tumor necrosis factor production	4	3.06E-04
KEGG Pathway	hsa05134	Legionellosis	3	1.60E-02
KEGG Pathway	hsa04145	Phagosome	4	1.71E-02
KEGG Pathway	hsa04640	Hematopoietic cell lineage	3	3.74E-02
KEGG Pathway	hsa04915	Estrogen signaling pathway	3	4.92E-02

**Table 3:** The hub genes in the turquoise and blue module.

Module	Gene Symbol	Gene Title	*p*-value	IC	MM
Turquoise	FCN1	ficolin (collagen/fibrinogen domain containing) 1	6.49E-07	25	0.96
Turquoise	S100A9	S100 calcium binding protein A9	4.26E-07	25	0.96
Turquoise	IGSF6	immunoglobulin superfamily, member 6	3.20E-04	21	0.95
Turquoise	HCK	HCK proto-oncogene, Src family tyrosine kinase	6.80E-05	18	0.96
Turquoise	CD14	CD14 molecule	2.10E-05	17	0.95
Turquoise	TLR2	toll-like receptor 2	4.54E-08	17	0.95
Turquoise	VCAN	versican	6.21E-08	17	0.96
Turquoise	PTAFR	platelet-activating factor receptor	1.40E-05	16	0.95
Turquoise	GLT1D1	glycosyltransferase 1 domain containing 1	6.57E-07	15	0.95
Turquoise	MS4A6A	membrane-spanning 4-domains, subfamily A, member 6A	4.00E-06	15	0.95
Blue	ABCA5	ATP-binding cassette, sub-family A (ABC1), member 5	2.24E-02	24	0.96
Blue	LUC7L	LUC7-like (S. cerevisiae)	1.78E-02	23	0.97
Blue	RBM6	RNA binding motif protein 6	4.01E-03	12	0.95
Blue	FAM134B	family with sequence similarity 134, member B	6.32E-03	11	0.96
Blue	CLUAP1	clusterin associated protein 1	8.74E-04	9	0.96
Blue	GABPB2	GA binding protein transcription factor, beta subunit 2	8.30E-03	8	0.94

## Data Availability

The microarray data were deposited into NCBI-GEO database under the accession numbers GSE19339, GSE48060, GSE66360, GSE97320, and GSE123487, the hyperlink to the dataset: https://www.ncbi.nlm.nih.gov/geo/query/acc.cgi?acc=GSE19339, https://www.ncbi.nlm.nih.gov/geo/query/acc.cgi?acc=GSE48060, https://www.ncbi.nlm.nih.gov/geo/query/acc.cgi?acc=GSE66360, https://www.ncbi.nlm.nih.gov/geo/query/acc.cgi?acc=GSE97320, https://www.ncbi.nlm.nih.gov/geo/query/acc.cgi?acc=GSE123487.

## Abbreviations

AMI: Acute myocardial infarction
ST2: IL-1RL-1, Interleukin 1 receptor-like 1
MiRNAs: MicroRNAs
WGCNA: Weighted Gene Coexpression Network Analysis
DEGs: Differentially expressed genes
GEO: Gene Expression Omnibus
RMA: Robust Multichip Analysis
DE: Differential expression
QC: Quality control
IQC: Internal homogeneity of coexpression structure among studies
EQC: External consistency of coexpression pattern with pathway database
AQCg: Accuracy of differentially expressed gene detection
CQCg: Consistency of differentially expressed gene detection
AQCp: Accuracy of enriched pathway identification
CQCp: Consistency of enriched pathway identification
PCA: Principal component analysis
FDR: False discovery rate
GO: Gene Ontology
KEGG: Kyoto Encyclopedia of Genes and Genomes
DAVID: Database for Annotation, Visualization and Integrated Discovery
TOM: Topological overlap matrix
ME: Module eigengene
MM: Module membership
GS: Gene significance
MS: Module significance
IC: Intramodular connectivity
3'-UTR: 3'-untranslated region
P_CT_: Probability of conserved targeting
SMR: Standardized mean rank
FCN1: Ficolin (collagen/fibrinogen domain containing) 1
S100A9: S100 calcium binding protein A9
IGSF6: Immunoglobulin superfamily, member 6
HCK: HCK protooncogene, Src family tyrosine kinase
CD14: CD14 molecule
TLR2: Toll like receptor 2
VCAN: Versican
PTAFR: Platelet-activating factor receptor
GLT1D1: Glycosyltransferase 1 domain containing 1
MS4A6A: Membrane-spanning 4-domains, subfamily A, member 6A
ABCA5: ATP-binding cassette, subfamily A (ABC1), member 5
LUC7L: LUC7-like (S. cerevisiae)
RBM6: RNA binding motif protein 6
FAM134B: Family with sequence similarity 134, member B
CLUAP1: Clusterin associated protein 1
GABPB2: GA binding protein transcription factor, beta subunit 2
ALDH2: Mitochondrial aldehyde dehydrogenase 2
BCL6: B-cell CLL/lymphoma 6
DUSP6: Dual specificity phosphatase 6
KCTD12: Potassium channel tetramerization domain containing 12
SFXN2: Sideroflexin 2
4-HNE: 4-hydroxynonenal
MDA: Malonaldehyde
HMOX1: Heme oxygenase (decycling) 1
S100A12: S100 calcium binding protein A12.

## Supplementary Materials

Supplementary Materials Supplementary Figure 1. Picking up a soft-threshold approximating to a scale-free topology criterion based on R square and mean connectivity. Supplementary Figure 2. Number of detected DEGs under different* q*-value threshold. Supplementary Figure 3. Correlation coefficient and significant* p*-value between each module and AMI.
