# Supplementary material for: Identification of Candidate Genes and MicroRNAs for Acute Myocardial Infarction by Weighted Gene Coexpression Network Analysis
Source: Biomed Res Int. 2019 Feb 11;2019:5742608. doi: 10.1155/2019/5742608 (PMC6388335; doi:10.1155/2019/5742608)
Supplement: Supplementary Materials — Supplementary Figure 1. Picking up a soft-threshold approximating to a scale-free topology criterion based on R square and mean connectivity. Supplementary Figure 2. Number of detected DEGs under different q-value threshold. Supplementary Figure 3. Correlation coefficient and significant p-value between each module and AMI. [file 5742608.f1.pdf]

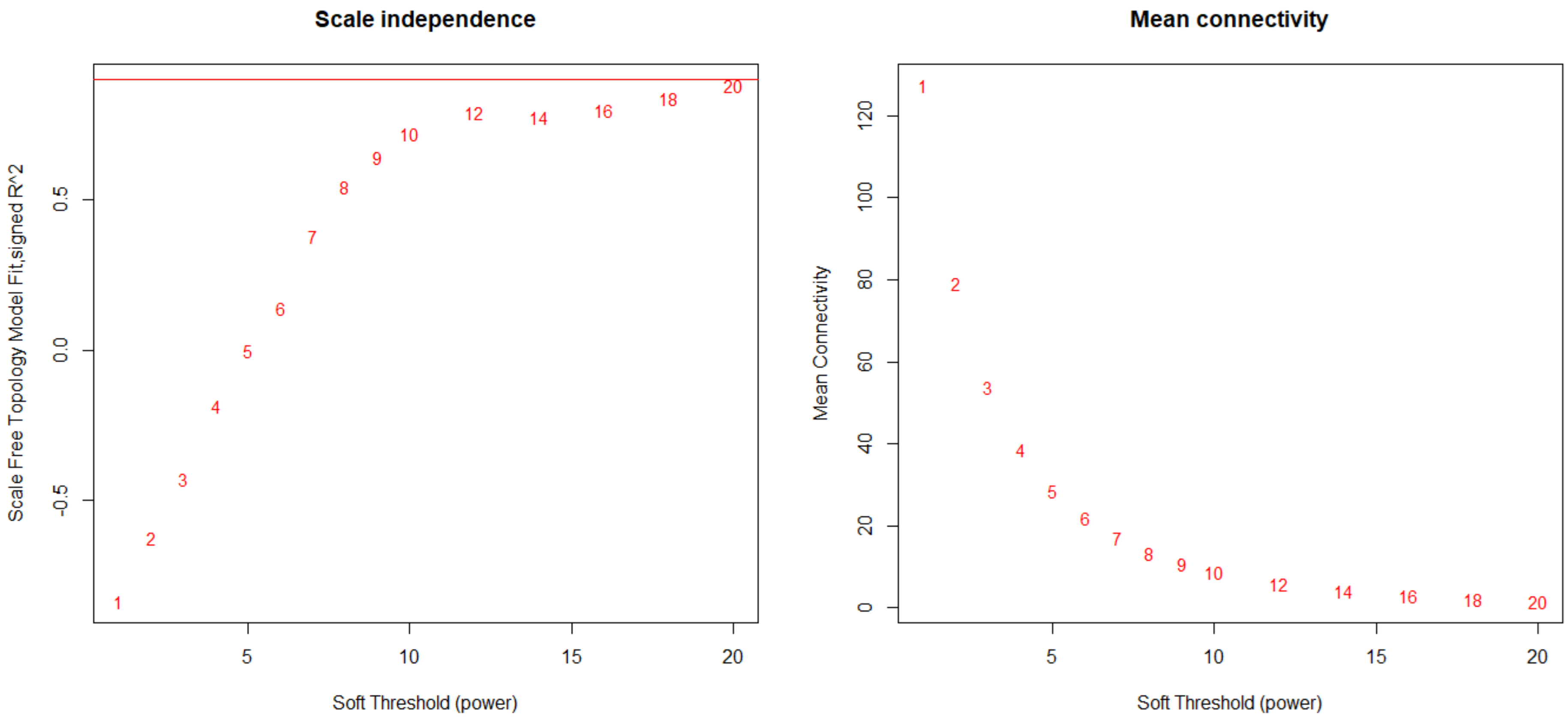

Supplementary Figure 1. Picking up a soft-threshold approximating to a scale free topology criterion based on R square and mean connectivity

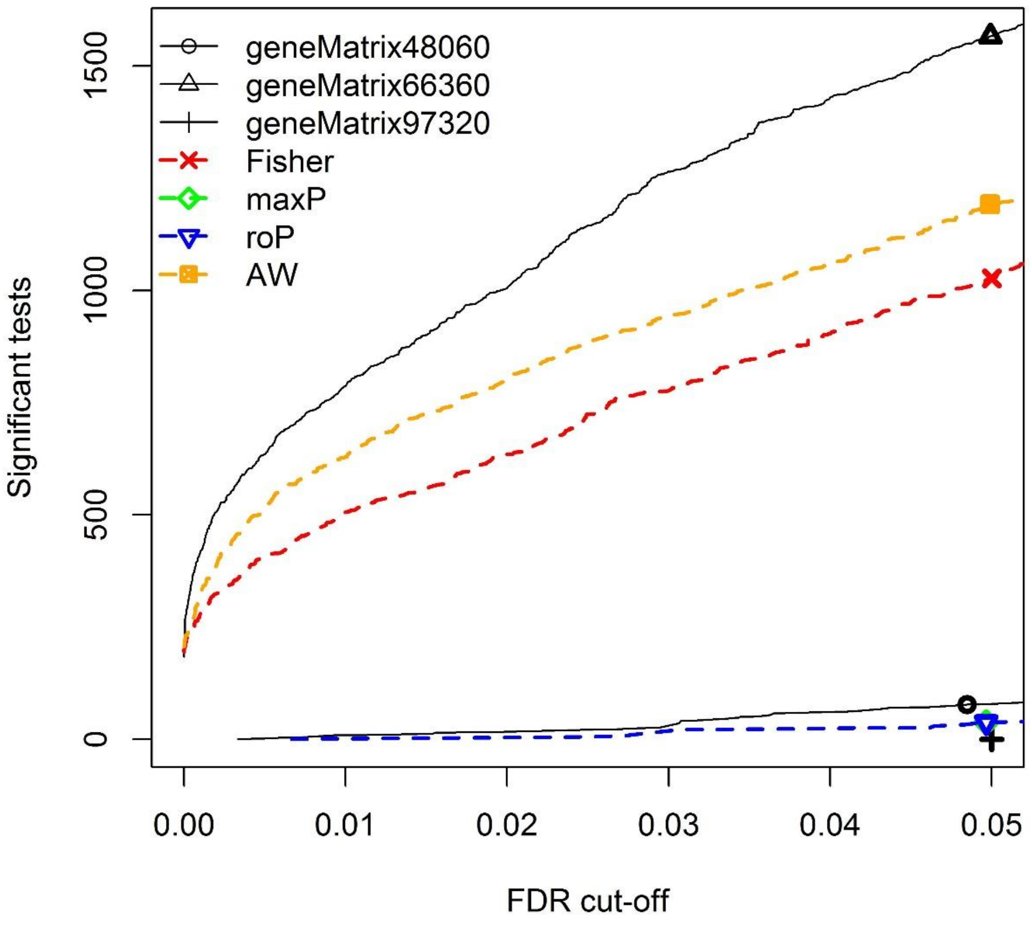

Supplementary Figure 2. Number of detected DEGs under different *q*-value threshold

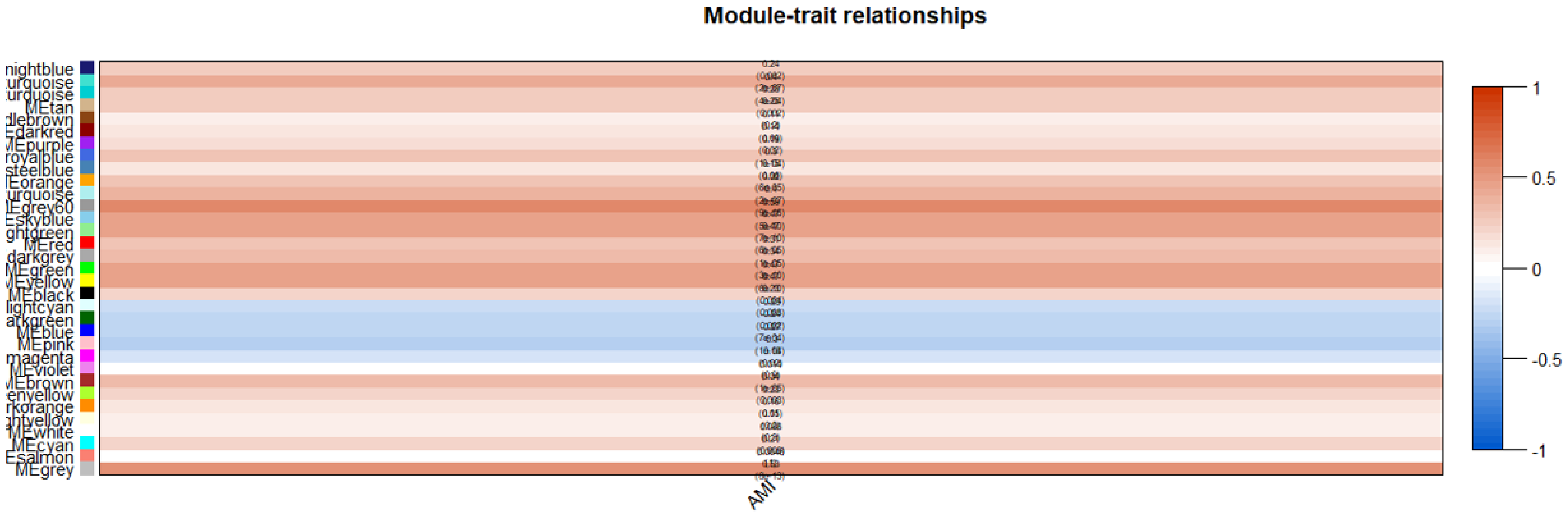

Supplementary Figure 3. Correlation coefficient and significant *p*-value between each module and AMI
